# Genotypic characteristics of Uropathogenic *Escherichia coli* isolated from complicated urinary tract infection (cUTI) and asymptomatic bacteriuria—a relational analysis

**DOI:** 10.7717/peerj.15305

**Published:** 2023-06-20

**Authors:** Lalitha Maniam, Kumutha Malar Vellasamy, Teng Aik Ong, Cindy Shuan Ju Teh, Kartini Abdul Jabar, Vanitha Mariappan, Vallikkannu Narayanan, Jamuna Vadivelu, Vinod Pallath

**Affiliations:** 1 Department of Medical Microbiology, Faculty of Medicine, Universiti Malaya, Kuala Lumpur, Malaysia; 2 Department of Surgery, Faculty of Medicine, Universiti Malaya, Kuala Lumpur, Malaysia; 3 Centre of Toxicology and Health Risk Studies (CORE), Faculty of Health Sciences, Universiti Kebangsaan Malaysia, Kuala Lumpur, Malaysia; 4 Department of Obstetrics and Gynaecology, Faculty of Medicine, Universiti Malaya, Kuala Lumpur, Malaysia; 5 Medical Education Research and Development Unit (MERDU), Faculty of Medicine, Universiti Malaya, Kuala Lumpur, Malaysia

**Keywords:** Uropathogenic *Escherichia coli*, Complicated urinary tract infection, Asymptomatic bacteriuria, Extraintestinal pathogenic *E. coli*, Pyelonephritis, Cystitis, Urosepsis, Recurrent urinary tract infection, Catheter associated urinary tract infection, Urinary tract infection disease severity

## Abstract

**Background:**

Uropathogenic *Escherichia coli* (UPEC) is the predominant agent causing various categories of complicated urinary tract infections (cUTI). Although existing data reveals that UPEC harboured numerous virulence determinants to aid its survival in the urinary tract, the reason behind the occurrence of differences in the clinical severity of uninary tract infections (UTI) demonstrated by the UPEC infection is poorly understood. Therefore, the present study aims to determine the distribution of virulence determinants and antimicrobial resistance among different phylogroups of UPEC isolated from various clinical categories of cUTI and asymptomatic bacteriuria (ASB) *E. coli* isolates. The study will also attempt a relational analysis of the genotypic characteristics of cUTI UPEC and ASB *E. coli* isolates.

**Methods:**

A total of 141 UPEC isolates from cUTI and 160 ASB *E. coli* isolates were obtained from Universiti Malaya Medical Centre (UMMC). Phylogrouping and the occurrence of virulence genes were investigated using polymerase chain reaction (PCR). Antimicrobial susceptibility of the isolates to different classes of antibiotics was determined using the Kirby Bauer Disc Diffusion method.

**Results:**

The cUTI isolates were distributed differentially among both Extraintestinal Pathogenic *E. coli* (ExPEC) and non-ExPEC phylogroups. Phylogroup B2 isolates were observed to possess the highest average aggregative virulence score (7.17), a probable representation of the capability to cause severe disease. Approximately 50% of the cUTI isolates tested in this study were multidrug resistant against common antibiotics used to treat UTI. Analysis of the occurrence of virulence genes among different cUTI categories demonstrated that UPEC isolates of pyelonephritis and urosepsis were highly virulent and had the highest average aggregative virulence scores of 7.80 and 6.89 respectively, compared to other clinical categories. Relational analysis of the occurrence of phylogroups and virulence determinants of UPEC and ASB *E. coli* isolates showed that 46.1% of UPEC and 34.3% of ASB *E. coli* from both categories were distributed in phylogroup B2 and had the highest average aggregative virulence score of 7.17 and 5.37, respectively. The data suggest that UPEC isolates which carry virulence genes from all four virulence genes groups studied (adhesions, iron uptake systems, toxins and capsule synthesis) and isolates from phylogroup B2 specifically could predispose to severe UTI involving the upper urinary tract. Therefore, specific analysis of the genotypic characteristics of UPEC could be further explored by incorporating the combination of virulence genes as a prognostic marker for predicting disease severity, in an attempt to propose a more evidence driven treatment decision-making for all UTI patients. This will go a long way in enhancing favourable therapeutic outcomes and reducing the antimicrobial resistance burden among UTI patients.

## Introduction

Urinary tract infections (UTIs) are among the most common bacterial infections, affecting around 150 million people annually worldwide (Chegini et al., 2021; Maniam et al., 2022). Despite current advances in healthcare management, the incidence of both community and healthcare-associated UTIs remains higher and causes a significant burden on the management and healthcare expenditure every year (Mann et al., 2017). Complicated urinary tract infections (cUTI) are the UTI category associated with heterogeneous complicated comorbidities or conditions such as anatomical and functional abnormalities of the urinary tract, coexistence with immunosuppressing diseases, indwelling urinary catheter, pregnancy, being male in gender and recurrent infection (Lodise et al., 2022; Sabih & Leslie, 2021). In comparison with uncomplicated UTIs which are easy to be resolved with antibiotics, the treatment of cUTI often poses significant failure rates of up to 27% (Eliakim-Raz et al., 2019). In many circumstances, cUTI often results in recurrent infections, extended hospitalization and urosepsis which may lead to fatality (Sabih & Leslie, 2021).

According to the European Association of Urology (EAU) urological infection guidelines, cUTI can be grouped into a few categories such as cystitis, pyelonephritis, urosepsis, recurrent UTI, and catheter-associated UTI (Bonkat et al., 2017). In this study, the cUTI cases which could not be clinically classified into these five clinical categories were grouped into cUTI with co-morbidities. This category of cUTI with co-morbidities represents general cUTI isolates that could probably cause any of the defined cUTI clinical manifestations. Treating UTIs with antibiotics is common in practice. However, the emergence of antimicrobial resistance (AMR) among the causative agents will hamper the desired treatment outcome (Dadgostar, 2019). The prescription of broad-spectrum antibiotics further enhances the emergence of multidrug resistance (MDR) organisms in the community (Kot, 2019).

Although various Gram-negative and Gram-positive bacterial species are known to cause all types of UTIs, the strains of *Escherichia coli* (*E. coli*) are the predominant agent responsible for more than 85% of all UTI categories (Barber et al., 2016; Davari Abad, Khameneh & Vahedi, 2019). *E. coli*, based on the outcome of its association with the host, can be categorized into three groups such as commensals, intestinal pathogenic *E. coli* (InPEC), and extraintestinal pathogenic *E. coli* (ExPEC) which causes UTI. A subgroup of this ExPEC associated with UTIs is known as uropathogenic *E. coli* (UPEC), which is very diverse in its pathogenic capability. UPEC harbours various virulence determinants such as adhesin (*fimH, papC, sfa, afa*), siderophore (*iroN*, *fyuA*, *iutA*, *chuA*), toxins (*cnf*, *hly*, *sat*, *vat*, *usp*) and capsule synthesis (*kpsMTII*, *ompT*, *traT*); encoded in different combinations in its pathogenicity islands (PAI), mobile genetic elements and plasmids (Dadi et al., 2020; Rezatofighi, Mirzarazi & Salehi, 2021; Tanabe et al., 2022). The acquisition of these virulence genes was observed to play an important role in the pathogenesis of UTI (Terlizzi, Gribaudo & Maffei, 2017). Typing the bacterial pathogen is the utmost important aspect in understanding the epidemiology of a disease and enabling diagnosis and prognosis of infectious diseases and their management (Kotłowski et al., 2020). Clermont’s *E. coli* phylogrouping scheme is widely reported for isolates from various niches and hosts across literature, and was able to easily classify the *E. coli* strains in different groups which were shown to be congruent with results from multilocus sequence typing (MLST) (Beghain et al., 2018; Clermont et al., 2013). Phylogenetically, *E. coli* can be categorized into seven phylogroups; namely A, B1, B2, C, D, E, F and cryptic clade 1 (CC1) Clermont et al., 2013). *E. coli* isolated from UTI mostly belong to phylogroups B2 and D and to a lesser extent to phylogroups A and B1 (Tanabe et al., 2022). Analysis of the phylogroups combined with analysis of the occurrence of virulence genes in these phylogroups are expected to provide insights to pathogenicity of the UPEC, leading to better treatment decisions and outcomes.

Asymptomatic bacteriuria (ASB) represents bacterial colonization in the urinary tract without causing any clinical symptoms to the host (Maniam et al., 2022; Roos et al., 2006). Patients with ASB may carry the bacteria in their urinary tract, mimicking the communalistic existence with the host. The occurrence of ASB strains in the urinary tract was known to benefit the host by preventing the colonization of virulent strains (Stork et al., 2018). ASB state is not treated in healthy individuals except for pregnant women and patients with urological procedures. Data from the literature have revealed that ASB isolates also belonged to ExPEC phylogroups and possessed various UPEC-associated virulence genes such as *fimH*, *papC*, *iroN*, *chuA*, *kpsMTII*, *ompT*, and *usp*. The presence of the UPEC-associated virulence genes among ASB *E. coli* isolates indicate a greater risk of developing symptomatic infections in persons with ASB (Maniam et al., 2022).

The incidence of UTI’s severity followed by developments of its associated complications were often associated with the anatomical site of infection, the integrity of the host defense mechanism and also pathogen-related factors in terms of virulence nature and MDR potential of the organism involved (Tarchouna et al., 2013). Early population-based studies on molecular characterization of UPEC reveal that isolates of UPEC are highly heterogeneous in their virulence potential and the possession of these genes was often shown to increase its fitness in the urinary tract (Lara et al., 2017; Rezatofighi, Mirzarazi & Salehi, 2021; Schifano et al., 2019). Relational analysis of the occurrence of virulence genes in relation to phylogroups and cUTI clinical categories are not reported in the literature.

This study intended to determine (i) the distribution of virulence determinants and AMR among different phylogroups of UPEC isolated from clinically manifesting UTI cases; and (ii) relational analysis of the occurrence of phylogroups, virulence determinants and MDR UPEC isolates from different cUTI categories and ASB *E. coli* isolates. The findings may reveal the differential virulence potential of the UPEC isolated from various UTI manifestations and ASB *E. coli.* An explicit understanding of the virulence potential of UPEC isolates from different cUTI categories may aid in the development of new diagnostic algorithms, leading to better management and prognosis.

## Materials & Methods

### Ethics approval

Ethical approval for this study was obtained from the Universiti Malaya Medical Centre (UMMC) Medical research ethics committee (MRECID 201765-5313). All the cUTI *E. coli* isolates and ASB *E. coli* isolates used in this study were obtained between July 2017 and October 2019 from the Medical Microbiology Diagnostic Laboratory, Urology ward and Obstetrics and Gynecology Department of UMMC. Written informed consent was obtained before sample collection from patients. Ethical approval also included the collection of diagnosed and reported isolates of *E. coli* from Medical Microbiology Diagnostic Laboratory. UPEC isolates were grouped into cUTI clinical categories such as cystitis, pyelonephritis, urosepsis, recurrent UTI, and catheter associated-UTI (CAUTI) based on reported clinical evaluation. Isolates with undetermined cUTI category were grouped into a category ‘cUTI with co-morbidities’. This category ‘cUTI with co-morbidities’ represents possible isolates that could cause any of the five defined clinical category manifestations. This was done to ensure the inclusion of all the clinical isolates in the study.

### Identification of cUTI UPEC isolates

All the bacterial isolates were further confirmed using standard biochemical tests (Odonkor & Ampofo, 2013). *E. coli* isolates were stored frozen in Luria-Bertani (LB) (HiMedia, Maharashtra, India) broth containing 30% glycerol at −70 °C until further investigation. The identification was confirmed further by detecting the presence of 16s rRNA in *E. coli* isolates using conventional polymerase chain reaction (PCR) (Huijsdens et al., 2002).

### Phylogenetic analysis

Bacterial DNA was extracted using the boiling method (Tewawong et al., 2020). Isolates were assigned to one of the eight phylogenetic groups of *E. coli* (A, B1, B2, C, D, E, F, CC1) by the quadruplex PCR assay as described by Clermont et al. (2013).

### Identification of virulence genes

A conventional PCR technique was used to detect 12 virulence genes that are associated with UPEC type 1 fimbriae (*fimH),* P fimbriae (*papC*), F1 C fimbriae (*sfa*), salmochelin receptor (*iroN*), heme receptor (*chuA*), yersiniabactin receptor (*fyuA*), cytotoxic necrotizing factor (*cnf*), secreted autotransporter protein (*sat*), hemolysin (*hlyC*), outer membrane protein T (*ompT*), kps M group II capsule (*kpsMTII*) and uropathogen specific protein (*usp*), using specific primers (Table S1) (Ejrnæs, 2011; Idress et al., 2010; Lara et al., 2017; Momtaz et al., 2013; Shreya Basu, Hazra & Mukherjee, 2013; Tabasi et al., 2016; Yun et al., 2014). PCR was performed in a 25 µL reaction volume containing 5 µL of PCR master mix 5X (Promega, Madison, WI, USA), 18 µL of RNA-free water, 0.5 µL of forward and reverse primer (10 µmol) (Integrated DNA Technologies, Singapore), and 1 µL of template DNA. The PCR amplification was performed using the following conditions; initial denaturation at 95 °C for 5 min, 25 cycles of denaturation at 94 °C for 30 s, annealing at respective gene’s temperature (Table S1) for 30 s, extension at 68 °C for 3 min and final extension at 72 °C for 10 min (CFX, 96, BioRad, Hercules, CA, USA). PCR products stained with SYBR safe dye (Invitrogen, Waltham, MA, USA) were resolved on 1.5% agarose gels by electrophoresis at 120 V for 60 min. On completion of the electrophoresis, the gel was visualized under UV-induced fluorescence.

### Antibiotic sensitivity test

Kirby-Bauer disc diffusion method was used to determine the antimicrobial susceptibility profile of UPEC isolates used in this study. The methods and conditions were exactly the same as previously described in the work by Maniam et al. (2022). Results obtained were interpreted according to Clinical Laboratory Standards International (CLSI) guidelines (CLSI, 2018). The antibiotics used for testing were: ampicillin (AMP) 10 µg, amikacin (AK) 30 µg, amoxicillin/clavulanate (AMC) 20/10 µg, ampicillin/sulbactam (SAM) 10/10 µg, aztreonam (ATM) 30 µg, cefuroxime (CXM) 30 µg, cefoxitin (FOX) 30 µg, cefotaxime (CTX) 30 µg, cefepime (FEP) 30 µg, ceftriaxone (CRO) 30 µg, imipenem (IPM) 10 µg, gentamicin (CN) 30 µg, norfloxacin (NOR) 30 µg, and piperacillin/tazobactam (TP) 100/10 µg. *E. coli* ATCC 25922 was used as the quality control strain in antimicrobial susceptibility determination (Shahbazi et al., 2018). A strain showing resistance to at least three antibiotic classes tested was considered an MDR strain (Magiorakos et al., 2012).

### Phylogrouping, virulence gene detection and antibiotic susceptibility test of ASB *E. coli* isolates

The same methodology adopted for UPEC isolates was also used for phylogrouping, virulence gene detection and antibiotic susceptibility testing of ASB *E. coli* isolates, which was reported in another preliminary report published by the research group (Maniam et al., 2022).

### Statistical analysis

Statistical analyses were performed using IBM Statistical Package for Social Sciences (SPSS) software (version 25.0). The Kruskal–Wallis test was used for the analysis of the occurrence of virulence determinants and antibiotic resistance among phylogroups of UPEC. Analysis of the occurrence of virulence determinants among different clinical categories was also performed through Kruskal–Wallis test. The Pearson Chi-square test was used to perform a comparison for the occurrence of virulent determinants between ExPEC with non-ExPEC phylogroups of UPEC and for overall comparison between UPEC and ASB *E. coli* isolates. Bonferroni adjusted *p* value for multiple comparisons was used to identify the significant differences in occurrences.

The aggregative virulence score for the occurrence of virulence genes in this study was calculated by averaging the number of virulence genes identified in each strain among the phylogroups, virulence functional groups and cUTI categories (Rezatofighi, Mirzarazi & Salehi, 2021). The co-occurrence analysis of the virulence genes was performed using pair-wise correlation using the Phi coefficient (Johnson et al., 2001a; Tabasi et al., 2016).

## Results

### Phylogenetic analysis of cUTI UPEC isolates

**Figure 1:**
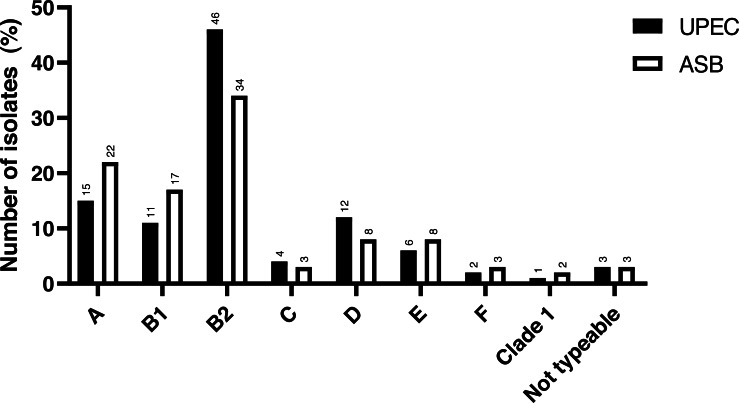
Distribution of phylogroups among cUTI UPEC and ASB *E. coli* isolates. Data for ASB *E. coli* isolates adapted and representation obtained from Maniam et al. (2022).

The study included a total of 141 UPEC isolates obtained. The phylogenetic analysis revealed that the majority of the cUTI UPEC isolates were from phylogroup B2 (46.1%), followed by group A (14.9%), group D (12.0%), group B1 (11.3%), group E (5.7%), group C (4.3%), group F (2.2%) and CC1 (0.7%). There were only four isolates (2.9%) which were non-typeable (NT) as per the classification proposed by Clermont et al. (2013). The comparison of the distribution of phylogroups between cUTI UPEC and ASB *E. coli* is depicted in Fig. 1.

### Distribution of virulence genes among phylogroups

Considering the cUTI UPEC isolates as a whole, the occurrence of the *fimH* (87.9%) gene was observed highest followed by *usp* (58.9%), *kpsMTII* (50.4%), *fyuA* (44.7%), *hlyA* (41.8%), *papC* (37.4%), *iroN* (36.2%), *ompT* (31.9%), *cnf* (29.8%), *sfa* (25.5%) and *sat* (12.8%) in descending order (Table 1). Analysis of the distribution of virulence genes among phylogroups revealed that UPEC from phylogroups B2 and E harboured all 12 virulence genes tested in this study in different permutations and combinations. It was observed that, there were significant differences (*p* value <0.004) for the occurrence of virulence genes among UPEC of ExPEC (B2, D, E and F) and non-ExPEC (A, B1 and C) for *fyuA*, *chuA*, *kpsMTII* and *usp* (Table 2). Analysis of the distribution of total virulence genes screened revealed that 43 (30.5%) isolates harboured at least three of the virulence genes tested, followed by 53 (37.6%) with four to six virulence genes and 29 (20.6%) with seven to nine virulence genes. There were 16 isolates of UPEC identified with 10 to 12 genes tested and the majority of these isolates (14 isolates) were from phylogroup B2. With regard to each of the virulence genes tested, there were significant differences identified in the presence of virulence genes among phylogroups for genes *sfa*, *papC, fyuA*, *chuA*, *ompT*, *kpsMTII* and *usp*. The occurrence of these genes was observed significantly higher among UPEC from phylogroup B2 (Table 1).

### Antibiotic susceptibility test results

UPEC isolated from different UTI manifestations were tested against 14 antibiotics representing nine antimicrobial classes. Resistance observed was highest against AMP (85.8%), followed by CTX (66.7%), AMC (63.1%), AK (60.3%), GN (53.9), TP (51.1), FEP (49.6%), CXM (43.3%), SAM (41.1%), IPM (39.7), NOR (32.6%), CRO (31.9%) and ATM (29.1%) (Table 3). Among the antibiotics tested in this study, resistance to FOX was observed to be the lowest (12.1%). There was no statistical difference observed for the occurrence of antibiotic resistance among phylogroups. Of the 141 UPEC, 123 (87.2%) isolates exhibited resistance to at least one antimicrobial tested, while 52.5% of UPEC were identified as MDR.

**Table 1:** Distribution of uropathogenic *Escherichia coli* (UPEC) virulence genes among different phylogroups, *n* (%).

**Virulence genes**	**A**	**B1**	**B2**	**C**	**D**	**E**	**F**	**CC1**	**NT**	**Total**	*p*-value
	21	16	65	6	17	8	3	1	4	141	
**Adhesion**											
*fimH*	15 (71.4)	15 (93.8)	60 (92.3)	6 (100)	14 (82.4)	7 (87.5)	2 (66.7)	1 (100)	4 (100)	124 (87.9)	0.245
*sfa*	3 (14.3)^ab^	3 (18.8)^ab^	27 (41.5)^a^	0^c^	0^c^	1 (12.5)^ab^	1 (33.3)^ab^	0^c^	1 (25.0)^ab^	36 (25.5)	0.013^*^
*papC*	5(23.8)^ab^	5 (31.0)^ab^	38 (57.9)^a^	0^c^	5 (29.4)^ab^	2 (25.0)^ab^	0^c^	0^c^	1 (25.0)^ab^	56 (37.4)	0.029^*^
**Siderophore**											
*iroN*	7 (33.3)	2 (12.5)	30 (46.2)	3 (50.0)	4 (23.5)	1 (12.5)	2 (66.7)	0	2 (50.0)	51 (36.2)	0.134
*fyuA*	4 (19.0)^b^	2 (12.5)^b^	42 (64.6)^a^	2 (33.3)^ab^	9 (52.9)^ab^	2 (25.0)^ab^	0^b^	0^b^	2 (50.0)^ab^	63 (44.7)	<0.001^*^
*chuA*	0^b^	0^b^	65(100)^a^	0^b^	17 (100)^a^	8 (100)^a^	8 (100)^a^	0^b^	0^b^	98 (69.4)	<0.001^*^
**Toxin**											
*cnf*	1 (4.8)^b^	2(13.3)^b^	32 (49.3)^a^	2 (33.3)^ab^	2(11.8)^b^	1 (12.5)^ab^	0^b^	0^b^	2 (50.0)^a^	42 (29.8)	<0.001^*^
*hlyA*	7 (33.3)	6 (37.5)	33 (50.8)	3 (50.0)	4 (23.5)	1 (12.5)	2 (66.7)	0	3 (75.0)	59 (41.8)	0.179
*sat*	1 (4.8)	0	10 (15.4)	1 (16.7)	3 (17.6)	2 (25.0)	0	0	1 (25.0)	18 (12.8)	0.582
*usp*	6 (28.6)^b^	6 (37.5)^b^	58 (89.2)^a^	2 (33.3)^b^	5 (29.40)^b^	5 (62.5)^ab^	0^b^	0^b^	1 (25.0)^b^	83 (58.9)	<0.001^*^
**Capsule synthesis**											
*ompT*	6 (28.6)	3 (18.8)	25 (38.5)	2 (33.3)	2 (11.8)	3 (37.5)	3 (100)	0	1 (25.0)	45 (31.9)	0.118
*kpsMTII*	4 (19.0)^b^	0^b^	47 (72.3)^a^	1 (16.7)^b^	12 (70.6)^a^	4 (50.0)^ab^	1 (33.3)^ab^	0^b^	2 (50.0)^ab^	71 (50.4)	<0.001^*^

**Notes.**

Group with different lowercase alphabets superscripted are statistically different based on pairwise comparison. Kruskal–Wallis test was used for statistical comparison and *p*-value <0.05^*^ was considered as significant. Significance values have been adjusted by the Bonferroni correction for multiple comparisons.

**Table 2:** Comparison of virulence gene occurrence among the cUTI UPEC isolates and ASB *E. coli* isolates, (*n*) (%).

**Virulence genes**	**cUTI UPEC ExPEC** **phylogroups** ***n* (%)**	**cUTI UPEC non- ExPEC phylogroups *n* (%)**	***p* value**	**UPEC** ***n* (%)**	**ASB***n* (%)	*p* value
*fimH*	84 (67.7)	40 (32.3)	0.464	124 (87.9)	121 (75.6)	0.006
*sfa*	29 (80.6)	7 (19.4)	0.041	36 (25.5)	27 (16.9)	0.066
*papC*	45 (80.4)	11 (19.6)	0.005	56 (37.4)	33 (20.6)	<0.001^*^
*iroN*	37 (72.5)	14 (27.5)	0.265	51 (36.2)	39 (24.4)	0.026
*fyuA*	53 (84.1)	10 (15.9)	<0.001^*^	63 (44.7)	51 (31.9)	0.022
*chuA*	94 (95.9)	4 (5.1)	<0.001^*^	98 (69.4)	88 (55.0)	0.010
*cnf*	35 (83.3)	7 (16.7)	0.006	42 (29.8)	36 (22.5)	0.150
*hlyA*	40 (67.8)	19 (32.2)	0.809	59 (41.8)	44 (27.5)	0.009
*sat*	16 (88.9)	2 (11.1)	0.032	18 (12.8)	12 (7.5)	0.128
*usp*	68 (81.9)	15 (18.1)	<0.001^*^	83 (58.9)	55 (34.4)	0.001^*^
*ompT*	33 (73.3)	12 (26.7)	0.250	45 (31.9)	24 (15.0)	0.001^*^
*kpsMTII*	65 (91.5)	6 (8.5)	<0.001^*^	71 (50.4)	61 (38.1)	0.033

**Notes.**

Chi-square test was used for statistical comparison and *p*-value has been adjusted by the Bonferroni correction for multiple comparisons (*p* < 0.004^*^).

**Table 3:** Antimicrobial resistance pattern observed among different phylogroups of cUTI UPEC, *n* (%).

**Antibiotics**	**Phylogroups**										*p*-value
	**A**	**B1**	**B2**	**C**	**D**	**E**	**F**	**CC 1**	**NT**	**Total**	
	21	16	65	6	17	8	3	1	4	141	
AMP	18 (85.7)	13 (81.3)	55 (84.6)	5 (83.3)	16 (94.1)	7 (87.5)	3 (100)	0	4 (100)	121 (85.8)	0.384
AMC	15 (71.4)	10 (62.5)	36 (55.4)	2 (33.3)	13 (76.5)	7 (87.5)	3 (100)	0	3 (75.0)	89 (63.1)	0.155
SAM	9 (42.9)	8 (50.0)	24 (36.9)	1 (16.7)	8 (47.1)	2 (25.0)	3 (100)	0	3 (75.0)	58 (41.1)	0.221
TP	9 (42.9)	8 (50.0)	33 (50.8)	3 (50.0)	12 (70.6)	2 (25.0)	3 (100)	0	2 (50.0)	72 (51.1)	0.325
CXM	8 (38.1)	7 (43.8)	30 (46.2)	2 (33.3)	7 (41.2)	2 (25.0)	3 (100)	0	2 (50.0)	61 (43.3)	0.622
FOX	0	3 (18.8)	7 (10.8)	1 (16.7)	3 (17.6)	2 (25.0)	0	0	1 (25.0)	17 (12.1)	0.528
CTX	14 (66.7)	10 (62.5)	45 (69.2)	4 (66.7)	11 (64.7)	4 (50.0)	3 (100)	0	3 (75.0)	94 (66.7)	0.803
FEP	8 (38.1)	9 (56.3)	34 (52.3)	4 (66.7)	6 (35.3)	3 (37.5)	3 (100)	0	3 (75.0)	70 (49.6)	0.335
CRO	6 (28.6)	5 (31.3)	20 (30.8)	3 (50.0)	5 (29.4)	1 (12.5)	3 (100)	0	2 (50.0)	45 (31.9)	0.266
ATM	5 (23.8)	6 (37.5)	17 (26.2)	2 (33.3)	5 (29.4)	1 (12.5)	3 (100)	0	2 (50.0)	41 (29.1)	0.203
IPM	5 (23.8)	5 (31.3)	32 (49.2)	2 (33.3)	5 (29.4)	2 (25.0)	2 (66.7)	0	3 (75.0)	56 (39.7)	0.231
AK	16 (76.2)	11 (68.8)	35 (53.8)	4 (66.7)	9 (52.9)	4 (50.0)	3 (100)	0	3 (75.0)	85 (60.3)	0.391
GN	9 (42.9)	9 (56.3)	37 (56.9)	3 (50.0)	8 (47.1)	4 (50.0)	3 (100)	0	3 (75.0)	76 (53.9)	0.676
NOR	9 (42.9)	6 (37.5)	18 (27.7)	2 (33.3)	5 (29.4)	2 (25.0)	2 (66.7)	0	2 (50.0)	46 (32.6)	0.823
MDR	11 (52.4)	8 (50.0)	37 (50.0)	3 (50.0)	7 (41.2)	2 (25.0)	3 (100)	0	3 (75.0)	74 (52.5)	–
Non-MDR	10 (47.6)	8 (50.0)	37 (50.0)	3 (50.0)	10 (58.8)	6 (75.0)	0	1 (100)	1 (25.0)	67 (47.5)	–

**Notes.**

MDR Multidrug resistance Non-MDR Non multidrug resistance

ampicillin (AMP,10µg), amoxicillin/clavulanate (AMC, 20/10µg), ampicillin/sulbactam (SAM, 10/10 µg), piperacillin /tazobactam (TP, 100/10µg), cefuroxime (CXM, 30µg), cefoxitin (FOX, 30µg), cefotaxime (CTX, 30µg), cefepime (FEP, 30µg), ceftriaxone (CRO, 30µg), aztreonam (ATM, 30µg), imipenem (IMP, 10µg), amikacin (AK, 30µg), gentamicin (CN, 30µg) and norfloxacin (NOR, 30µg).

Kruskal–Wallis test was used for statistical comparison and *p*-value <0.05 was considered as significant. Significance values have been adjusted by the Bonferroni correction for multiple comparisons.

**Table 4:** Distribution of phylogroups, virulence genes, multidrug resistance of UPEC isolates according to different cUTI categories; *n* (%).

	**Complicated UTI categories**							
	**Cystitis**	**Pyelonephritis**	**Urosepsis**	**Recurrent UTI**	**Catheter-associated UTI**	**cUTI with co-morbidities**	**Total**	*p*-value
	**18 (12.8)**	**10 (7.1)**	**8 (5.7)**	**14 (9.9)**	**40 (28.4)**	**51 (36.2)**	**141 (100)**	
A	1 (5.6)	1 (10.0)	0	3 (21.4)	7 (17.5)	9 (17.6)	–	–
B1	2 (11.1)	0	1 (12.5)	1 (7.1)	5 (12.5)	7 (13.7)	–	–
B2	11 (61.1)	9 (90.0)	5 (62.5)	4 (28.6)	17 (42.5)	19 (37.3)	–	–
C	1 (5.6)	0	0	0	3 (7.5)	2 (3.9)	–	–
D	2 (11.1)	0	1 (12.5)	3 (21.4)	4 (10.0)	7 (13.9)	–	–
E	1 (5.6)	0	1 (12.5)	2 (14.3)	1 (2.5)	3 (5.9)	–	–
F	0	0	0	0	1 (2.5)	2 (3.9)	–	–
CC1	0	0	0	0	1 (1.6)	1 (1.9)	–	–
Not typeable				1 (7.1)	1(1.6)	1 (1.9)	–	–
*fimH*	16 (88.9)	10 (100)	7 (87.5)	11 (78.6)	35 (87.5)	45 (88.2)	124 (87.9)	0.770
*sfa*	4 (22.2)	4 (40.0)	2 (25.0)	2 (14.3)	9 (22.5)	15 (29.4)	36 (25.5)	0.774
*papC*	7 (38.9)^bc^	9 (90.0)^a^	6 (75.0)^ab^	7 (50.0)^ab^	16 (40.0)^bc^	11 (21.6)^c^	56 (39.7)	<0.001^*^
*iroN*	3 (16.9)	6 (60.0)	5 (62.5)	5 (35.7)	13 (32.5)	19 (37.3)	51 (36.2)	0.155
*fyuA*	7 (38.9)	5 (50.0)	4 (50.0)	6 (42.9)	17 (42.5)	24 (47.1)	63 (44.7)	0.985
*chuA*	14 (77.8)	9 (90.0)	7 (87.5)	10 (71.4)	25 (62.5)	33 (64.7)	98 (69.5)	0.386
*cnf*	4 (22.8)^b^	7 (70.0)^a^	6 (75.0)^a^	3 (21.4)^b^	11 (27.5)^b^	11 (21.6)^b^	42 (29.8)	0.003^*^
*hlyA*	6 (33.3)	6 (60.0)	6 (75.0)	6 (42.9)	13 (32.5)	22 (43.1)	59 (41.8)	0.226
*sat*	2 (11.1)	0	1 (12.5)	2 (14.3)	4 (10.0)	9 (17.6)	18 (12.8)	0.718
*usp*	14 (77.8)	9 (90.0)	3 (37.5)	8 (57.1)	9 (22.5)	24 (27.1)	45 (31.9)	0.058
*ompT*	5 (27.8)	3 (30.0)	5 (62.5)	7 (50.0)	21 (52.5)	17 (33.3)	71 (50.4)	0.305
*kpsMTII*	8 (44.4)	7 (70.0)	6 (75.0)	6 (42.9)	26 (65.0)	23 (45.1)	83 (58.9)	0.718
MDR	13 (72.2)	9 (90.0)	5 (62.5)	7 (50.0)	17 (42.5)	23 (45.1)	74 (52.5)	–
Non-MDR	5 (27.8)	1 (10.0)	3 (37.5)	7 (50.0)	23 (57.5)	28 (54.9)	67 (47.5)	–

**Notes.**

Group with different lowercase alphabets superscripted are statistically different based on pairwise comparison. Kruskal–Wallis test was used for statistical comparison and *p*-value <0.05^*^ was considered as significant. Significance values have been adjusted by the Bonferroni correction for multiple comparisons.

### Distribution of phylogroups, virulence genes and multidrug resistance among different cUTI categories

The data on the distribution of phylogroups, virulence genes, and MDR of UPEC isolated from different cUTI manifestations are depicted in Table 4. Analysis of the distribution of phylogroups among UPEC of various cUTI categories demonstrated a common trend of higher distribution of isolates from phylogroup B2. Among the cUTI categories, the occurrence of phylogroup B2 was observed highest among isolates of pyelonephritis, followed by urosepsis and cystitis. Isolates of CAUTI, and recurrent UTI exhibited higher diversity in the distribution of phylogroups, especially the occurrence of isolates from phylogroups C, E, F, and CC1. The assembled cUTI with co-morbidities category also showed the same trend with high diversity with the presence of isolates from all the phylogroups except for CC1.

Data from the analysis on the distribution of virulence genes among UPEC from different cUTI categories revealed that isolates from cystitis, urosepsis, recurrent UTI and CAUTI (also cUTI with co-morbidities category) harbour all virulence genes tested in this study. Isolates from pyelonephritis cases notably lacked *sat* gene. Of the different cUTI categories included, isolates of pyelonephritis and urosepsis showed higher occurrence for all the virulence genes tested in this study. UPEC isolates from pyelonephritis showed the highest occurrence for *fimH* (100%), *sfa* (100%), *papC* (90.0%), *chuA* (90.0%), *usp* (90.0%) and *fyuA* (50.0%). While, UPEC isolated from urosepsis exhibited the highest occurrence of *iroN* (62.5%), *cnf* (75.0%), *hlyA* (75.0%), *ompT* (62.5%) and *kpsMTII* (70.0%). The distribution of these virulence genes among the *other* cUTI categories such as cystitis, recurrent UTI and CAUTI (also cUTI with co-morbidities category) were moderate to low in comparison with isolates of pyelonephritis and urosepsis. Overall, statistical differences in the distribution of virulence genes were observed for *papC* (*p* < 0.006) and *cnf* (*p* < 0.007) genes among UPEC of different cUTI categories.

Analysis of the occurrence of MDR UPEC among the different clinical categories revealed that 90% of pyelonephritis isolates were MDR, followed by 72.0% of cystitis isolates, 62.5% of urosepsis isolates, 50% of recurrent UTI isolates, and 42.5% of CAUTI isolates in the descending order. The assembled cUTI with co-morbidities category also had 45.1% MDR isolates.

### Relational analysis of the occurrence of phylogroups and virulence determinants among UPEC isolates from different cUTI categories and ASB *E. coli* isolates

Table 5 shows an analysis of aggregative virulence score for UPEC and ASB *E. coli* isolates according to phylogroups. Results obtained from this study demonstrate that 38.3% of UPEC isolates possessed a minimum of four to a maximum of six virulence genes, followed by 29.8% of strains with a minimum of one to a maximum of three virulence genes, 20.6% of strains with a minimum of seven to a maximum of nine virulence genes and 11.3% of strains with a minimum of 10 to a maximum of 12 virulence genes in different permutations and combinations. Analysis of average aggregative virulence scores of UPEC from different phylogroups revealed that isolates from phylogroup B2 had the highest aggregative virulence score (7.17), and there were 14 isolates of these phylogroups possessing a minimum of 10 virulence genes. Lower average aggregative virulence score was observed for UPEC isolates from phylogroup A (2.90) and B1 (2.81) and most of the isolates from these phylogroups only possessed a maximum of three virulence genes.

**Table 5:** Average aggregative virulence scores of cUTI UPEC and ASB *E. coli* isolates among the phylogroups, *n* (%).

**Total number of virulence genes**	**Phylogroups**																			
	**A**		**B1**		**B2**		**C**		**D**		**E**		**F**		**CC1**		**NT**		**Total**	
	**UPEC**	**ASB**	**UPEC**	**ASB**	**UPEC**	**ASB**	**UPEC**	**ASB**	**UPEC**	**ASB**	**UPEC**	**ASB**	**UPEC**	**ASB**	**UPEC**	**ASB**	**UPEC**	**ASB**	**UPEC**	**ASB**
	*n* = 16	*n* = 35	*n* = 16	*n* = 27	*n* = 65	*n* = 55	*n* = 6	*n* = 5	*n* = 17	*n* = 13	*n* = 8	*n* = 13	*n* = 3	*n* = 5	*n* = 1	*n* = 3	*n* = 4	*n* = 4	*n* = 141	*n* = 160
0 to 3	15	18	11	15	5	35	3	3	3	9	4	5	0	2	0	1	1	3	42 (29.8)	90 (56.3)
4 to 6	4	9	5	7	20	13	3	2	13	2	3	5	3	2	1	2	2	1	54 (38.3)	43 (26.9)
7 to 9	2	9	0	5	25	5	0	0	1	2	0	2	0	0	0	0	0	0	29 (20.6)	23 (14.4)
10 to 12	0	0	0	0	14	2	0	0	0	0	1	1	0	1	0	0	1	0	16 (11.3)	4 (2.4)
**Average Aggregative virulence score**	2.90	2.13	2.81	2.11	7.17	5.37	3.83	3.60	4.53	3.64	4.63	3.36	3.61	4.40	2.00	2.67	5.75	1.75	–	–

**Notes.**

Average aggregative virulence scores were calculated by averaging the total number of virulence genes identified in each strain among the phylogroups.

In comparison with cUTI isolates, ASB *E. coli* isolates were identified to possess a reduced number of virulence genes (Table 5). Statistical analysis of the occurrence of virulence genes among cUTI and ASB *E. coli* isolates using the Chi-square test revealed that, there were statistical differences in the occurrence of virulence genes among these isolates for the occurrence of three virulence genes tested (*papC*: *p* < 0.001, *ompT*: *p* < 0.001, and *usp*; *p* < 0.001) (Table 2). It was observed that 56.3% of ASB *E. coli* isolates possessed zero to a maximum of three virulence genes, followed by 26.9% with a minimum of four to a maximum of six virulence genes, 14.4% strains with a minimum of seven to a maximum of nine virulence genes and only four strains were harbouring a minimum of 10 to a maximum of 12 virulence genes in different permutations and combinations. Similar to UPEC isolates, ASB *E. coli* isolates from phylogroup B2 were also observed with the highest average aggregative virulence score (5.37), which was lower than the average aggregative virulence scores of phylogroup B2 cUTI UPEC isolates.

The analysis of comparison of average aggregative virulence score among UPEC from different cUTI categories revealed that UPEC isolated from pyelonephritis had the highest score (7.80), followed by urosepsis (6.87), cystitis (5.00), recurrent UTI (5.00), and CAUTI (4.70). The assembled cUTI with the comorbidities category had an average aggregative virulence score of 4.80 (Table 6). It was observed that 57.1% of UPEC isolated from recurrent UTI, 45.0% UPEC from CAUTI and 41.2% of UPEC from cUTI with co-morbidities harboured a minimum of one to a maximum of three virulence genes tested in this study in different combinations. In comparison with UPEC from the above mentioned cUTI categories, 83.9% cystitis isolates were observed to harbour four to six virulence genes tested, followed by 60.0% of pyelonephritis isolates and 37.5% of urosepsis isolates with seven to nine virulence genes, respectively.

**Table 6:** Average aggregative virulence scores of cUTI UPEC isolates based on clinical categories, *n* (%).

**Total virulence genes**	**cUTI categories**					
	**Cystitis**	**Pyelonephritis**	**Urosepsis**	**Recurrent UTI**	**Catheter-associated UTI**	**cUTI with co-morbidities**
	**18 (12.8)**	**10 (7.1)**	**8 (5.7)**	**14 (9.9)**	**40 (28.4)**	**51 (36.2)**
0 to 3	2 (11.1)	0	2 (25.0)	8 (57.1)	18 (45.0)	21 (41.2)
4 to 6	15 (83.9)	1 (10.0)	1 (17.5)	2 (14.3)	12 (30.0)	17 (33.3)
7 to 9	1 (5.5)	6 (60.0)	3 (37.5)	2 (14.3)	5 (12.5)	8 (15.7)
10 to 12	0	3 (30.0)	2 (25.0)	2 (14.3)	5 (12.5)	5 (9.8)
Average aggregative virulence score	5.00	7.80	6.89	5.00	4.71	4.80

**Notes.**

Average aggregative virulence scores were calculated by averaging the total number of virulence genes identified in each strain of the cUTI categories.

The analysis of the average aggregative virulence score for the individual virulence functional groups (adhesin, iron uptake, toxin and capsule synthesis) is depicted in Table 7. Data demonstrated that UPEC isolates of pyelonephritis and urosepsis have higher average aggregative virulence score for all the virulence functional categories tested in this study. Isolates of pyelonephritis and urosepsis possessed a minimum of two to three virulence genes from each virulence category tested in this study. In comparison with UPEC strains, ASB *E. coli* isolates were identified to have lower average aggregative virulence scores for each of the virulence functional categories included in this study. However, it was also observed that there were few UPEC isolates of other UTI categories (cystitis, recurrent UTI, CAUTI, and UTI with comorbidities), which did not possess any of the tested virulence genes encoding for a particular virulence functional group. The number of ASB isolates with no virulence genes in some of the functional groups was higher than that of UPEC isolates.

**Table 7:** Comparison of virulence gene occurrence and average aggregative virulence score in cUTI UPEC and ASB *E. coli* isolates based on functional virulence category, *n* (%).

**Functional Virulence category**	**Cystitis** (18)	**Pyelonephritis** (10)	**Urosepsis** (8)	**Recurrent UTI** (14)	**Catheter associated UTI (40)**	**UTI with co-morbidities** (51)	**Overall UPEC isolates (141)**	**ASB*E. coli*** (160)
**Adhesion**								
0 gene	1 (5.56)	0	0	3 (21.43)	5 (12.50)	3 (5.88)	12 (8.51)	29 (18.13)
1 gene	7 (38.89)	1 (10.00)	2 (25.00)	4 (28.57)	15 (37.50)	30 (58.82)	59 (41.84)	91 (56.88)
2 genes	10 (55.56)	5 (50.00)	5 (62.50)	5 (35.71)	15 (37.50)	13 (25.49)	53 (37.59)	30 (18.75)
3 genes	0	4 (40.00)	1 (12.50)	2 (14.29)	5 (12.50)	5 (9.80)	17 (12.06)	10 (6.25)
AVS	1.50	2.30	1.88	1.43	1.50	1.39	1.53	1.13
**Iron uptake system**							
0 gene	4 (22.22)	0	0	4 (28.57)	7 (17.50)	12 (23.53)	29 (20.57)	49 (30.63)
1 gene	6 (33.33)	2 (20.00)	2 (25.00)	4 (28.57)	16 (40.00)	11 (21.57)	39 (27.66)	54 (33.75)
2 genes	6 (33.33)	3 (30.00)	3 (37.50)	1 (7.14)	12 (30.00)	19 (37.25)	46 (32.62)	47 (29.38)
3 genes	2 (11.11)	5 (50.00)	3 (37.50)	5 (35.71)	5 (12.50)	9 (17.65)	27 (19.15)	10 (6.25)
AVS	1.33	2.20	2.00	1.50	1.38	1.49	1.50	1.11
**Toxin**								
0 gene	0	1 (10.00)	0	5 (35.71)	7 (17.50)	13 (25.49)	26 (18.44)	68 (42.50)
1 gene	10 (55.56)	1 (10.00)	1 (12.50)	5 (35.71)	19 (47.50)	20 (39.22)	56 (39.72)	55 (34.38)
2 genes	8 (44.44)	3 (30.00)	3 (37.50)	1 (7.14)	8 (20.00)	10 (19.61)	33 (23.40)	19 (11.88)
3 genes	0	5 (50.00)	4 (50.00)	2 (14.29)	5 (12.50)	7 (13.71)	23 (16.31	18 (11.25)
4 genes	0	0	0	1 (7.14)	1 (2.50)	1 (1.96)	3 (2.13)	0
**AVS**	1.44	2.20	2.38	1.21	1.35	1.27	1.44	0.92
**Capsule synthesis**								
0 gene	8 (44.44)	3 (30.00)	2 (25.00)	3 (21.43)	18 (45.00)	18 (35.29)	52 (36.88)	83 (51.88)
1 gene	7 (38.89)	4 (40.00)	4 (50.00)	7 (50.00)	14 (35.00)	26 (50.98)	62 (43.97)	69 (43.13)
2 genes	3 (16.67)	3 (30.00)	2 (25.00)	4 (28.57)	8 (20.00)	7 (13.73)	27 (19.15)	8 (5.00)
**AVS**	0.72	1.00	1.00	1.07	0.75	0.78	0.82	0.53

**Notes.**

0 gene: absence of any tested virulence gene in the specific functional group, 1 gene: one gene is present among the tested three genes in the specific functional group, 2 genes: two genes are present among the tested three genes in the specific functional group etc., AVS- average aggregative virulence score.

### Co-occurrence analysis for the occurrence of virulence genes among cUTI UPEC and ASB *E. coli* isolates

The co-occurrence analysis of the occurrence of virulence genes among cUTI UPEC isolates, revealed a strong positive correlation (*r* > 0.5) between *sfa* and *cnf* (*r* = 0.508), *iroN* with *hlyA* (*r* = 0.858), and *chuA* with *kpsMTII* (*r* = 0.513) (Table 8). A moderate positive correlation (0.4 <*r* < 0.5) was noticed for *fyuA* and *kpsMTII* (*r* = 0.493).

**Table 8:** Co-occurrence of virulence genes among cUTI UPEC and ASB *E. coli* isolates.

		***fimH***	***sfa***	***papC***	***iroN***	***fyuA***	***chuA***	***cnf***	***hlyA***	***sat***	***ompT***	***kpsMTII***	***usp***
*fimH*	UPEC	1	.117	.167^*^	.052	.245^**^	.086	.241^**^	.093	.076	−.027	.068	.025
		ASB	1	.061	.074	−.118	.388^**^	−.104	−.043	−.042	.162^*^	.157^*^	.086	−.079
*sfa*	UPEC		1	.090	.372^**^	.194^*^	.176^*^	.508^**^	.295^**^	−.078	.192^*^	.191^*^	.227^**^
		ASB		1	.224^**^	.444^**^	.157^*^	.173^*^	.556^**^	.433^**^	−.128	−.049	.299^**^	.377^**^
*papC*	UPEC			1	.324^**^	.174^*^	.223^**^	.327^**^	.369^**^	−.050	.097	.139	.189^*^
		ASB			1	.178^*^	.314^**^	.088	.354^**^	.447^**^	.148	.089	.300^**^	.249^**^
*iroN*	UPEC				1	.303^**^	.114	.349^**^	.858^**^	−.155	.213^*^	.068	.032
	ASB				1	.049	.192^*^	.531^**^	.563^**^	−.162^*^	−.157^*^	.244^**^	.294^**^
*fyuA*	UPEC					1	.347^**^	.350^**^	.221^**^	.255^**^	.242^**^	.493^**^	.177^*^
	ASB					1	.187^*^	.145	.089	.314^**^	.051	.430^**^	.239^**^
*chuA*	UPEC						1	.263^**^	.062	.161	.090	.513^**^	.366^**^
	ASB						1	.187^*^	.135	−.029	.099	.425^**^	.443^**^
*cnf*	UPEC							1	.328^**^	−.063	.120	.275^**^	.252^**^
	ASB							1	.406^**^	−.153	.025	.317^**^	.398^**^
*hlyA*	UPEC								1	−.152	.159	−.020	.031
	ASB								1	−.122	−.141	.179^*^	.291^**^
*sat*	UPEC									1	.240^**^	.167^*^	−.009
	ASB									1	.013	.314^**^	−.206^**^
*ompT*	UPEC										1	.132	.026
	ASB										1	−.041	.028
*kpsMTII*	UPEC											1	.360^**^
	ASB											1	.543^**^
*usp*	UPEC												1
	ASB												1

**Notes.**

Significance levels:

** *p* ≤ 0.01.

* 0.01 < *p* < 0.05.

+ positive correlation.

– negative correlation.

The co-occurrence analysis performed for ASB *E. coli* isolates revealed similar strong positive correlations observed for *sfa* with *cnf* (*r* = 0.556) *iroN* with *hlyA* (*r* = 0.563), and *iroN* with *cnf* (*r* = 0.531) (Table 8). Moderate correlations were observed between *sfa* with *iroN* (*r* = 0.444), *sfa* with *hlyA* (*r* = 0.433), *papC* with *hlyA* (*r* = 0.447), *fyuA* with *kpsMTII* (*r* = 0.430), *chuA* with *kpsMTII* (*r* = 0.425), and *chuA* with *usp* (*r* = 0.433).

## Discussion

To the best of our knowledge, the present work is the very first study describing the occurrence of phylogroups, virulence genes and MDR potential of UPEC isolated from more than three cUTI categories (cystitis, pyelonephritis, urosepsis, recurrent UTI, and CAUTI). Complicated UTI with co-morbidities is a group of heterogeneous isolates, put together by the authors, as the patients they were isolated from were not clinically assigned to any specified cUTI category.

### Analysis of the occurrence of phylogroups

UPEC isolates in this study were differentially distributed among eight phylogroups. The phylogenetic distribution pattern of cUTI UPEC isolates was similar to that reported in other studies, with a greater occurrence of isolates from phylogroup B2 (Dadi et al., 2020; Halaji et al., 2022; Tewawong et al., 2020). A meta-analysis of 28 studies reporting UPEC virulence genes also identified a similar pattern (Halaji et al., 2022). Overall, 68.1% of UPEC isolates were from ExPEC-associated phylogroups, while 31.9% were from non-ExPEC. The present study showed that the UPEC isolates in traditional phylogroups (A, B1, B2 and D) were redistributed to Clermonts’ extended new phylogroups (C, E and F). These findings were also consistent with the meta-analysis report by Halaji et al. (2022). Phylogenetic analysis indicated that the occurrence of UPEC from non-EXPEC phylogroups was lower than reported in other studies (Gatya Al-Mayahie et al., 2022; Tanabe et al., 2022). It was observed that the distribution of UPEC into the NT group was also lower than the reported 27% from Iran (Iranpour et al., 2015).

Similar to the trends of phylogroups distribution among cUTI isolates, ASB *E. coli* isolates were also reported to be highly distributed among ExPEC representing phylogroups (53.78%) than non-ExPEC phylogroups (41.82%) (Maniam et al., 2022). Such similar higher occurrences of ASB *E. coli* from ExPEC representing phylogroup B2 were also reported by Amarsy et al. (2019) and Lavigne et al. (2011). The higher distribution of ASB *E. coli* in ExPEC phylogroups suggests that ASB strains have the potential to cause symptomatic UTI. Any treatment decisions regarding the ASB should involve a detailed evaluation of the virulence characteristics of these isolates in the best interest of favourable treatment outcome and prevention of future complications.

### Distribution of virulence genes among phylogroups

In parallel with other studies (Dadi et al., 2020; Er et al., 2015; Rezatofighi, Mirzarazi & Salehi, 2021; Tanabe et al., 2022), analysis of the distribution of virulence genes among phylogroups in this study revealed that cUTI UPEC isolates from phylogroup B2 had a higher presence of all the virulence genes tested. In comparison with isolates from non-ExPEC phylogroups, UPEC from ExPEC phylogroups B2 and D showed higher percentage distribution of all the virulence genes studied, particularly the occurrence of *sfa, papC, fyuA, cnf, usp* and *kpsMTII* genes. These findings were in agreement with the report by Belmont-Monroy et al. (2022), in which phylogroup B2 was shown to possess six to ten virulence genes, in comparison to non-ExPEC phylogroups possessing one to five virulence genes. Isolates from phylogroup E, which is a sub-group from D, was identified to harbour all the virulence genes tested. UPEC isolates from non-ExPEC phylogroups A, B1 and C were generally observed to possess a reduced number of virulence genes than the ExPEC phylogroups. Considering ExPEC and non-ExPEC phylogroups together, there were statistically significant differences in the presence of virulence genes *fyuA*, *chuA*, *usp* and *kpsMTII* (*p* < 0.001) (Table 2).

The analysis of the aggregative virulence score among phylogroups demonstrates that there is a potential association between the phylogroups and the virulence of the isolates. In both cUTI UPEC and ASB *E. coli* isolates, strains from phylogroup B2 demonstrated the highest average aggregative virulence scores of 7.17 and 5.37, respectively, than strains from other phylogroups. Similar higher average aggregative virulence scores of 9.00 and 5.95 for UPEC from phylogroup B2 was reported by Kudinha et al. (2013) and Rezatofighi, Mirzarazi & Salehi (2021), respectively. Data obtained from the analysis of average aggregative virulence scores among the strains of cUTI UPEC and ASB *E. coli* isolates revealed that *E. coli* isolates of ExPEC, particularly isolates of B2 were highly virulent, possessing a greater number of virulence genes than strains from non-ExPEC phylogroup.

Data across the literature has demonstrated that ASB *E. coli* isolates of various phylogroups were also possessing the virulence-associated genes and the presence of these genes were hypothesized to facilitate the survival of the bacterium in the urinary tract of the host (Eghbalpour et al., 2022; Maniam et al., 2022). Data from the previous study by Maniam et al. (2022) on genotypic characteristics of ASB *E. coli* isolates revealed that these isolates also carried all the virulence genes tested in this study in different permutations and combinations among its isolates. It was observed that the occurrence of these virulence genes were lower in frequency among all the phylogroups for ASB *E. coli* isolates. There were statistically significant differences observed in the overall occurence of virulence genes *fyuA*, *chuA*, *usp* and *kpsMTII* (*p* < 0.001) between UPEC and ASB *E. coli* isolates (Table 2).

### Relational analysis of the occurrence of virulence genes

In this study, the occurrence of *fimH* gene was also observed as the highest (number) among tested virulence genes for each cUTI category. Adhesin virulence factors of UPEC are crucial to facilitate the colonisation and initiation of the infection process, of which the occurrence of type 1 fimbriae (*fimH*) gene was identified as highly abundant and conserved among the isolates of *E. coli* of various phylogroups and pathotypes (Firoozeh et al., 2022; Norinder et al., 2012). The presence of *fimH* gene was known to promote the binding of this bacterium to mannosylated glycoproteins of uroplakin 1a which are abundant along the urothelium of the urinary bladder (Johnson et al., 2005b). Adhesin between UPEC and superficial facet cells in host urothelium was known to aid in the invasion of this bacterium into the cytoplasm, and thus promotes the production of intracellular bacterial communities (IBC) (Schwartz et al., 2013). This subsequently protects the bacteria from the sheer force of urination and the host’s innate immune system and promotes prolonged colonisation in the urinary tract. The highest occurrence of these genes among both cUTI and ASB *E. coli* indicate the importance of this gene as a vital element of survival of most *E. coli* isolates of various pathotypes. Although the occurrence of *fimH* was observed to be common among UPEC and commensal *E. coli*, the expression of this gene is partially regulated by phase variation of promoter gene (*fimS*), which eventually results in phase ON (expression) or OFF (non-expression) orientations (Firoozeh et al., 2022; Foroogh et al., 2021; Subashchandrabose & Mobley, 2015). However, the expression analysis of *fimH* and occurrence of *fimS* were not investigated in this study.

The presence of the S-fimbriae (*sfa*) gene is associated with UPEC’s ability to bind to the terminal of sialyl-galactoside residues of urothelium present on various parts of the kidney including tubules of glomeruli (Desvaux et al., 2020). Therefore, the occurrence of *sfa* gene is often associated with UPEC survival strategy in the upper urinary tract, in turn associating the gene with severe UTI categories of pyelonephritis and urosepsis. In this study, the distribution of *sfa* gene was observed among 40% of the isolates of pyelonephritis and 25% among isolates of urosepsis. The isolates of other cUTI categories were also observed to possess *sfa* gene with minimal frequency.

The occurrence of the p-fimbriae (*pap*) gene, was significantly associated with pyelonephritis. UPEC possessing *pap* gene will be able to bind to the Gal *α* (1–4) - Gal moieties (glycosphingolipids) located in the urothelial cells, especially in the kidney. Although the occurrence of *pap* gene was lower than other *fimH* genes, the highest occurrence of this gene was observed among pyelonephritis isolates. Higher distribution of *pap* gene (75%) was also observed among isolates of urosepsis, indicating the association of this gene with increased disease severity. The occurrence of *papC* genes among other cUTI isolates indicates the probability of the development of more severe diseases like pyelonephritis or urosepsis in these patients.

Iron is crucial for the survival of UPEC, required for various cellular and biological processes of nucleotide biosynthesis, electron transport and peroxidase reduction (Robinson, Heffernan & Henderson, 2018). However, iron is limiting in the hostile environment of the urinary tract. As part of its survival strategies, UPEC is able to synthesize various small and high-affinity molecules called siderophores, which enable them to scavenge ferric iron from host cells. Results from this study reveal that UPEC isolated from all UTI categories, were identified to possess varying distribution of salmochelin (*iroN*), yersiniabactin (*fyuA*) and heme receptor (*chuA*) genes. In addition to facilitating the iron acquisition, Gao et al. (2012) demonstrated that the acquisition of both *iroN* and *fyuA* genes could predispose UPEC to invade the urothelial, thus causing infection severity.

In addition to siderophore molecules, the isolates of UPEC were also able to scavenge the heme-bound iron. *Chu* is an important heme transport gene possessed mostly by UPEC, also known to facilitate the acquisition of iron to aid in bacterial survival. Similarly, the occurrence of *chuA* gene is reported to be associated with increased resistance of invading uropathogens against antimicrobial peptides and thus predisposes to biofilm formation, which could further enhance the fitness of UPEC in the urinary tract (Hancock & Klemm, 2007).

Toxins are known to be associated with UTI severity. UPEC possessing toxins are able to invade the host cells, allowing the bacterium to survive in a more quiescent state, promoting long-term bacterial persistence within the urinary tract (Dhakal & Mulvey, 2012). Hemolysin A (*hlyA*) gene encodes for pore-forming toxins, which increases the permeability of the cell membrane, causing cell death at high concentration (Wiles & Mulvey, 2013). As per the literature, the occurrence of *hlyA* is often associated with urosepsis and pyelonephritis, with an increased likelihood of renal complications later (Jahandeh et al., 2015).

Cytotoxic necrotising factor (*cnf*) gene in UPEC activates small Rho-family GTPases which promotes various events in the host cells, especially multinucleation and cell spreading, apoptosis of urothelium, decreasing polymorphonuclear leucocytes and also activation of nuclear factor, which could predispose to the disease severity. Acquisition of *cnf* and *hlyA* increases the chances of invasion of UPEC into urothelial cells of the kidney, which increases the risk of urosepsis. Based on the results from this study, the isolates from all cUTI categories possess these two virulence genes in varying distribution. However, a higher distribution of these genes was observed among isolates of urosepsis and pyelonephritis corresponding with its known disease severity. In contrast to the report by Dadi et al. (2020), which reported nearly 29% of the occurrence of *cnf* gene among pyelonephritis isolates, the occurrence of this gene in this study was remarkably high (70%). Although, a reduced occurrence of these genes was observed among the isolates of other cUTI categories such as cystitis, recurrent UTI, CAUTI and cUTI with comorbidities; the isolates with these genes may predispose patients to a higher risk of severe UTI (Vejborg et al., 2011).

The occurrence of secreted autotransporter protein (*sat*) was often found to be associated with cytotoxic effects on renal and bladder cells. In literature, the occurrence of *sat* gene was often associated with pyelonephritis, corresponding to the virulence potential of isolates possessing them (Guyer et al., 2002). In the present study, none of the pyelonephritis isolates and only one isolate of urosepsis was identified to harbour this gene. A lower distribution of this gene was also observed among the isolates of other cUTI categories, in contrast to the existing data on the prevalence of this gene among UPEC isolates (Morales-Espinosa et al., 2016).

The presence of protectins as polysaccharide capsules on the surface of UPEC is well known to aid UPEC attachment on various surfaces and protects the bacterium from the host immune system, bactericidal effects and also from antimicrobial agents (Sarkar et al., 2014). The occurrence of the outer membrane protein (*ompT*) gene among UPEC enables this bacterium to produce an enzyme with serine proteases activity, which helps the bacterium to cleave the cationic peptides with antibiotic activity. This subsequently promotes the bacteria to retain longer in the urinary tract by inhibiting the antimicrobial peptides produced by the host’s innate immune system. The occurrence of *ompT* gene in this study was observed to be highest among the isolates of urosepsis followed by CAUTI and recurrent UTI.

The distribution of another protectins, a capsule-producing gene, *kpsMTII* was also observed highly distributed among the isolates of both urosepsis and pyelonephritis. The occurrence of *kpsMTII* gene in the UPEC is known to play an important role in the development of IBC as well as exhibiting resistance to antimicrobial agents. The presence of protectins could enhance the survival of UPEC in bloodstream, necessitating its existence in urosepsis strains.

### Association of antimicrobial resistance among phylogroups

In most clinical circumstances, the inadequate treatment outcome from cUTI was often reported to be associated with increased MDR potential of the organism involved. The incidence of the emergence of MDR, especially in UPEC has become a major threat in UTI management. The data on antimicrobial resistance of UPEC isolated from cUTI in this study demonstrated higher resistance to all antimicrobials tested. Prior use of antibiotics, non-empirical antibiotic prescriptions, hospitalisation, age, genitourinary disturbances and recurrent infections (UTI) are known predisposing risk factors for emergence of MDR organisms (Kot, 2019). Data from the current study revealed that more than 40% of the isolates demonstrated resistance to nine antimicrobial agents tested (AMP, AMC, SAM, TP, CXM, CTX, FEP, AK, and GN). A similar trend was also reported by Malekzadegan et al. (2018) and Tewawong et al. (2020). MDR UPEC reported by the present study (52.5%) was lower than previous reports of 77.8% by Malekzadegan et al. (2018), 63.3% by Ramírez-Castillo et al. (2018) and 97% by Paniagua-Contreras et al. (2017). The MDR and challenges in the management of these cUTI conditions in all these study settings probably represents a wider global situation, warranting urgent attention. It was observed that the AMR of cUTI UPEC isolates were comparatively higher than the AMR among ASB *E. coli* isolates from the same study setting (Maniam et al., 2022). The occurrence of MDR among ASB isolates could be regarded as a bigger public health threat, indicating the spread of MDR strains in the community.

### Relational analysis of phylogroups, distribution of virulence genes and MDR among different cUTI categories and ASB *E. coli* isolates

UPEC are highly diverse pathotypes of ExPEC, identified to possess numerous virulence factors associated with adhesin, iron uptake system, toxin and capsule production among the isolates from various epidemiological backgrounds as reported in the literature (Firoozeh et al., 2022; Halaji et al., 2022; Jahandeh et al., 2015). The differences in clinical severity of cUTI are known to be multifactorial, depending on the virulence of the invading pathogen and the susceptibility of the host, especially if there is a concomitant urological illness. The diagnosis and treatment of any of the UTI categories depend on the clinical manifestations and a significant number of uropathogens isolated. Despite the known and well-studied association between the presence of virulence genes with UTI pathogenesis, the current diagnosis and prognosis of UTI still overlook the nature of the organism, including its virulence profile.

Based on the findings in this study, UPEC isolated from the five different cUTI categories (and the added category of cUTI with co-morbidities) were observed to be highly heterogeneous, possessing the virulence genes of adhesin, iron uptake system, toxin and capsule synthesis in different permutations and combinations. Acquisition of virulence genes from different functional groups could be collectively facilitating UPEC’s ability to survive in the hostile environment of the urinary tract. The present study also provides valid evidence towards the relationship between the number of virulence genes possessed and cUTI disease severity. It was observed that UPEC isolates of upper cUTI categories such as urosepsis and pyelonephritis had higher aggregative virulence scores than UPEC isolates of cystitis representing lower UTI category. Most of the UPEC isolates representing upper cUTI categories were identified to possess at least one virulence gene tested from each virulence functional category, demonstrating their increased fitness to survive in the upper urinary tract of the host. Studies by Ruiz et al. (2002) and Johnson et al. (2005a), have reported UPEC isolated from non-invasive UTI, such as cystitis had less virulence score than those isolates causing more-invasive UPEC syndromes such as pyelonephritis. However, reports on virulence score among UPEC isolated from recurrent UTI and CAUTI were not available in the existing literature.

Analysis of co-occurrence of the virulence genes revealed that the co-occurrence of *sfa/cnf* and *iroN/hlyA* are common among UPEC and ASB, indicating the probability of a common origin for the virulence determinants. The varying clinical manifestations of cUTI and ASB could be considered as diverging ends of the clinical spectrum of UTI, and the isolates associated with ASB could probably cause clinically manifesting UTI. Previous reports by Foxman et al. (1995) and Johnson et al. (2001b) have shown that association between virulence factors vary and genetic linkages between virulence genes were not constant, indicating the possibility of horizontal transfer of virulence genes. The findings of higher average aggregative virulence scores for isolates causing invasive UTIs (pyelonephritis and urosepsis) in comparison to ASB possibly provides evidences towards horizontal gene acquisition leading to enhanced virulence and survival capabilities among the ExPEC (Boyd & Hartl, 1998; Johnson, Russo & Donnenberg, 2018). A comprehensive genetic profiling based on virulence determinants could be a promising tool for determining prognostic probability of severe cUTI diseases.

On analysis, it was found that the isolates with two virulence genes and cause clinically manifesting UTI had adhesins and iron uptake system genes, adhesins and toxins or adhesins and capsule synthesis genes together. The isolates with three virulence genes had adhesins, iron uptake systems and toxins or adhesins, iron uptake systems and capsule synthesis genes in combination. As they progressed to possess four genes, the isolates had a combination of adhesins, iron uptake systems, toxins and capsule synthesis genes together. As the number of virulence genes increased from four to 12 genes, each of the isolates started demonstrating various genes of the four virulence gene groups (adhesins, iron uptake systems, toxins and capsule synthesis).

There were a few UPEC strains isolated from low-severity UTI diseases, such as cystitis, recurrent UTI and CAUTI, which closely mimicked the genetic profile of UPEC of high-severity diseases. These isolates indicate a prognostic probability of the risk of development of high severity diseases in these cUTI patients. Despite the occurrence of a higher number of virulence genes among some of the isolates from cystitis, recurrent UTI and CAUTI, the probable reason behind their reduced disease severity could be due to the lack of expression of the virulence genes involved. Although a few UPEC isolates (*n* = 42) of various cUTI categories, were identified with the minimal number (1 to 3) virulence genes tested in this study, the occurrence of these isolates in high cUTI disease severity could probably be due to possession of other UPEC associated virulence genes which were not screened in this study.

The isolates of UPEC causing cUTI in this study demonstrated a similar trend in terms of occurrence of phylogroups and virulence genes in comparison to ASB *E. coli* isolates as reported by Maniam et al. (2022), which is also from the same study setting as this study. However, in both *E. coli* pathotypes (cUTI and ASB), isolates from phylogroup B2 were observed higher in distribution and were found to possess a high number of virulence genes tested; thus, assumed to be highly pathogenic than isolates of other phylogroups. In a previous study by Maniam et al. (2022), the relational analysis of the occurrence of *papC/sfa, iroN/fyuA, cnf/hlyA, kpsMTII,* and *usp* virulence genes among ASB strains was recommended to be prognostic towards the cumulative virulence potential of the isolates in causing clinically significant UTI cases. In agreement with this, results from this study revealed that cUTI UPEC isolates from phylogroup B2 had significantly different occurrence for *fyuA*, *chuA*, *cnf*, *kpsMTII* and *usp*. This evidence could be explored further with appropriate sample sizes and study design to understand the cumulative effect of combination of genes in virulence potential of the phylogroups.

### Limitations of the study

The current study is explorative and relational in design and tested for the occurrence of the genes that were reported in the literature. There could be other virulence genes influencing the clinical outcome. The current study reports data based on a limited sample size if individual cUTI categories are considered. Due to non-comparable differences in the sample size for each cUTI category, the statistical comparison to check the significant association between the occurrences of virulence genes within the clinical categories could not be performed. Furthermore, the current study objective did not include the expression levels of virulence genes.

## Conclusions

The cUTI isolates were distributed differentially among both ExPEC and non-ExPEC phylogroups. Phylogroup B2 isolates were observed to possess the highest average aggregative virulence score (7.17). In addition to that, the occurrence of MDR strains was observed across all the phylogroups, indicating an alarming situation in the emergence of MDR strains across *E. coli* of various pathotypes representing pathogens (ExPEC) and commensals (non-ExPEC). The observations of higher resistance against the common antibiotics used to treat UTI and the isolates of B2 phylogroup causing severe cUTI demonstrating higher MDR could be considered as warning signs to carefully relook at the diagnostic algorithm UTIs. Including the genetic profile of the pathogen isolated in the diagnostic algorithm will enable prognosis towards probable disease development and will ensure a better management of the patients.

Similar to the UPEC isolates in this study, ASB *E. coli* isolates reported from the same study setting were also highly heterogeneous in their virulence profile. Although these ASB isolates were also mostly distributed in phylogroup B2, generally they were identified with a reduced number of virulence genes and lower frequency of MDR potential.

Analysis of the occurrence of virulence genes among UPEC isolates of various disease categories revealed that the occurrence of the virulence genes tested was not distinctive to any clinical cUTI categories. However, the data from the analysis of the average aggregative virulence score in this study indicated the probable relation between the increased cumulative actions of virulence genes with increased cUTI disease severity. The average aggregative virulence score of B2 phylogroup isolates from cUTI categories was higher than the average aggregative virulence score of B2 phylogroup isolates from ASB. The data strongly points towards the accumulation of virulence genes as a predicative indicator of disease severity. The strains with higher aggregative virulence scores could also be found among ASB isolates. This observation points towards possibility of higher expression of these genes among the isolates from clinically manifesting cUTI categories in comparison to ASB isolates, which could be explored further.

The data suggest that UPEC isolates which carry virulence genes from all four virulence genes groups studied (adhesins, iron uptake systems, toxins and capsule synthesis) and isolates from phylogroup B2 specifically could predispose to severe UTI categories involving the upper urinary tract. Therefore, specific exploration of the genotypic characteristics of UPEC could be further performed through analysis of the actions of combination of virulence genes, as a prognostic marker for predicting disease severity. Such an enhanced diagnostic algorithm which considers isolate characteristics will enable a more evidence driven treatment decision making, ensuring reduced complications and recurrence. This will go a long way in enhancing favourable therapeutic outcomes and reducing the antimicrobial resistance burden among UTI patients.

##  Supplemental Information

10.7717/peerj.15305/supp-1 Supplemental Information 1 Phylogroups, identification of virulence genes and MDR potential of UPEC isolates

10.7717/peerj.15305/supp-2 Supplemental Information 2 Virulence genes used for conventional PCR F: Forward primer, R: Reverse primer, Tm (° C) is the melting temperature used for the respective primer

## Additional Information and Declarations

### Data Availability

The following information was supplied regarding data availability: The raw data measurements are available in the Supplementary File.
