# Supplementary material for: Genotypic characteristics of Uropathogenic Escherichia coli isolated from complicated urinary tract infection (cUTI) and asymptomatic bacteriuria—a relational analysis
Source: PeerJ. 2023 Jun 20;11:e15305. doi: 10.7717/peerj.15305 (PMC10289082; doi:10.7717/peerj.15305)
Supplement: Supplemental Information 2 — F: Forward primer, R: Reverse primer, Tm (° C) is the melting temperature used for the respective primer [file peerj-11-15305-s002.docx]

**Table S1: Virulence genes used for conventional PCR**

| **Virulence Genes** | **Primer sequences (5’ to 3’)** | **Base pair (bp)** | **Tm (°C)** | **Reference** |
| --- | --- | --- | --- | --- |
| **Adhesion** |  |  |  |  |
| *papC* | F:GACGGCTGTACTGCAGGGTGTGGC  R:ATATCCTTTCTGCAGGGATGCAATA | 328 | 61 | (Basu et al., 2013) |
| *fimH* | F:TGCAGAACGGATAAGCCGTGG  R:GCAGTCACCTGCCCTCCGGTA | 508 | 54 | (Yun et al., 2014) |
| *Sfa* | F:CTCCGGAGAACTGGGTGCATCTTAC  R:CGGAGGAGTAATTACAAACCTGGCA | 410 | 59 | (Tabasi et al., 2016) |
| **Iron uptake** |  |  |  |  |
| *iroN* | F:AAGTCAAAGCAGGGGTTGCCCG  R:GACGCCGACATTAAGACGCAG | 665 | 59 | (Basu et al., 2013) |
| *fyuA* | F:TGAGTGGGAAATACACCACC  R:TTACCCGCATTGCTTAATGTC | 715 | 54 | (Lara et al., 2017) |
| **Toxin** |  |  |  |  |
| *hlyA* | F:AACAAGGATAAGCACTGTTCTGGCT  R:ACCATATAAGCGGTCATTCCCGTCA | 1177 | 61 | (Yun et al., 2014) |
| *cnf* | F:AAGATGGAGTTTCCTATGCAGGAG  R:TGGAGTTTCCTATGCAGGAG | 498 | 58 | (Ejrnæs et al., 2011) |
| *sat* | F:TATCACGCAATGCCAATGTT  R:GACCCGGCGTTACAGTTTTA | 393 | 63 | (Idress et al., 2010) |
| *usp* | F:ACATTCACGGCAAGCCTCAG R:GCGAGTTCCTGGTGAAAGC | 448 | 62 | (Momtaz et al., 2013) |
| **Capsule synthesis** | | | | |
| *kpsMTII* | F:GCGCATTTGCTGATACTGTTG  R:CATCCAGACGATAAGCATGAGCA | 272 | 54 | (Yun et al., 2014) |
| *ompT* | F:ATCTAGCCGAAGAAGGAGGC  R:CCCGGGTCATAGTGTTCATC | 559 | 59 | (Momtaz et al., 2013) |
|  |  |  |  |  |
| *16s rRNA* | F:CATGCCGCGTGTATGAAGAA  R:CGGGTAACGTCAATGAGCAAA | 100 | 59 | (Huijsdens et al., 2002) |

F: Forward primer, R : Reverse primer, Tm (˚C) is the melting temperature used for the respective primer
